# Honey bee (*Apis mellifera*) colony health and pathogen composition in migratory beekeeping operations involved in California almond pollination

**DOI:** 10.1371/journal.pone.0182814

**Published:** 2017-08-17

**Authors:** William Glenny, Ian Cavigli, Katie F. Daughenbaugh, Rosemarie Radford, Susan E. Kegley, Michelle L. Flenniken

**Affiliations:** 1 Department of Ecology, Montana State University, Bozeman, Montana, United States of America; 2 Pollinator Health Center, Montana State University, Bozeman, Montana, United States of America; 3 Department of Plant Sciences and Plant Pathology, Montana State University, Bozeman, Montana, United States of America; 4 Pesticide Research Institute, Berkeley, California, United States of America; University of North Carolina at Greensboro, UNITED STATES

## Abstract

Honey bees are important pollinators of agricultural crops. Pathogens and other factors have been implicated in high annual losses of honey bee colonies in North America and some European countries. To further investigate the relationship between multiple factors, including pathogen prevalence and abundance and colony health, we monitored commercially managed migratory honey bee colonies involved in California almond pollination in 2014. At each sampling event, honey bee colony health was assessed, using colony population size as a proxy for health, and the prevalence and abundance of seven honey bee pathogens was evaluated using PCR and quantitative PCR, respectively. In this sample cohort, pathogen prevalence and abundance did not correlate with colony health, but did correlate with the date of sampling. In general, pathogen prevalence (i.e., the number of specific pathogens harbored within a colony) was lower early in the year (January—March) and was greater in the summer, with peak prevalence occurring in June. Pathogen abundance in individual honey bee colonies varied throughout the year and was strongly associated with the sampling date, and was influenced by beekeeping operation, colony health, and mite infestation level. Together, data from this and other observational cohort studies that monitor individual honey bee colonies and precisely account for sampling date (i.e., day of year) will lead to a better understanding of the influence of pathogens on colony mortality and the effects of other factors on these associations.

## Introduction

Honey bees (*Apis mellifera*) are the primary insect pollinators of agricultural crops, including fruits, nuts, and vegetables, which have an approximate annual value of $17–18 billion in the United States [1,2]. California almond production is the most renowned example of the role of honey bee pollination services in the US. Every year, over 60% of the approximately 2.5 million commercially managed honey bee colonies in the United States are transported to the Central Valley of California to pollinate the almond crop. California almond growers produce ~ 80% of the global almond supply, which was valued at $1.7 billion in 2014 [3]. Honey bee colonies are important for the pollination of plants in both agricultural and non-agricultural landscapes [4,5]. Thus, high annual honey bee colony losses in the US, which have averaged ~ 33% annually since 2006 and increased from historic levels of approximately 12%, are concerning to stakeholders, including beekeepers, growers, and scientists [6,7]. To compensate for increased colony losses and meet pollination service demands, beekeeping operations have been splitting their colonies (i.e., producing two colonies from one colony) more frequently. Therefore, the total number of commercially managed honey bee colonies in the US has been maintained at approximately 2.5 million since the late 1990s [8] despite unsustainable annual colony losses.

There are multiple factors that impact colony health, including pathogens (i.e., mites, viruses, bacteria, and fungi), queen failure, colony genetics, weather, nutrition, management practices (i.e., transportation, treatments), and agrochemical exposure [7,9–18]. Honey bee colony health is typically evaluated by estimating colony population size [19–23]. Colonies are comprised of ~35,000 sterile female workers, hundreds of males (drones), and a single reproductive queen that can lay approximately 1,000 eggs per day [24]. While no single factor has been deemed responsible for high annual honey bee colony losses, pathogens are major contributing factors [6,7,10,11,21,22,25–33].

Honey bees host a diversity of pathogens, the majority of which are single-stranded RNA viruses [33,34]. Honey bee infecting viruses include Acute bee paralysis virus (ABPV), Black queen cell virus (BQCV), Deformed wing virus (DWV), Israeli acute paralysis virus (IAPV), Kashmir bee virus (KBV), Sacbrood virus (SBV), Chronic bee paralysis virus (CBPV) [34–38] and the Lake Sinai viruses [23]. In addition to viruses, pathogens of honey bees include eukaryotes such as the trypanosomatid *Lotmaria passim* (formerly *Crithidia mellificae* strain sf [39,40]), the microsporidial pathogen *Nosema ceranae* [41], and bacterial pathogens such as *Paenibacillus larvae* [42] and *Melissococcus plutonius* [43], the causative agents of American and European foulbrood diseases, respectively. In addition, the ectoparasitic mite, *Varroa destructor*, contributes to decreased colony health by feeding on developing bees (brood) and facilitating virus transmission [44] (i.e., DWV [14,45], KBV [46,47], and IAPV [34,44,48]). Mite parasitization of developing honey bees can result in physical deformities, reduced body weight, greater DWV virus levels, and/or death [44,49].

Temporal monitoring studies are essential for identifying and understanding the factors that influence honey bee colony health and pathogen prevalence and abundance. Identifying particular pathogens or suites of pathogens that are associated with colony losses is complicated because pathogen prevalence and abundance varies by season and geographic location [21,22,25,26,31]. Furthermore, there have been relatively few studies that have monitored individual commercially managed colonies [7,10,15–17,21,22]. The majority of studies in the US have monitored honey bee health at the apiary level with the main goals of detecting exotic pathogens and establishing a baseline understanding of honey bee colony health [6,7,15,17,30]. Additional studies that monitor colonies located in varying geographic locations are needed to better understand the impacts of multiple factors on honey bee colony health. To address this knowledge gap, we monitored Minnesota-based commercially managed honey bee colonies involved in the 2014 California almond pollination event. The goal of this study was to investigate the association between multiple factors, including pathogen seasonality, pathogen abundance, beekeeping operation, colony population size, and level of mite infestation, on honey bee colony health and colony losses. We determined that sampling date strongly influenced pathogen prevalence and abundance and confirmed a strong association between DWV abundance and *Varroa destructor* mite infestation. However, the abundance of other viruses (i.e., BQCV, LSV1, and LSV2) was not associated with mite infestation. Together, data from this and other temporal observational cohort studies that precisely account for sampling date (i.e., day of the year) and monitor individual honey bee colonies will lead to a better understanding of the association between factors affecting pathogen abundance and honey bee colony health.

## Methods

### Longitudinal monitoring and sampling of commercially managed honey bee colonies

**Fig 1:**
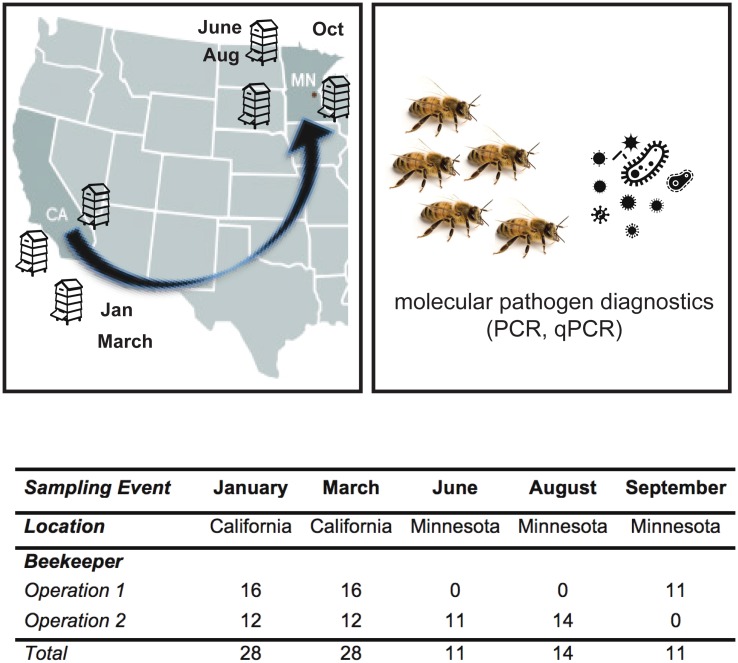
Commercially managed honey bee colonies were longitudinally monitored before, during, and after the 2014 almond pollination season. Honey bee colonies from two Minnesota-based commercial beekeeping operations that transport their colonies to California for almond pollination were monitored from January 2014 to January 2015. At each sampling event, colony health, using colony population size as a proxy for health, was monitored and samples of live honey bees were obtained. In a subset of samples described in the table, PCR was utilized to assess pathogen prevalence and qPCR was utilized to determine pathogen abundance.

Honey bee (*Apis mellifera*) colonies from two Minnesota-based commercial beekeeping operations that transport honey bee colonies to California for almond pollination were monitored for one year (i.e., from January 2014 to January 2015). At the onset of the study in January 2014, beekeepers identified 20 colonies that successfully overwintered. Samples from a subset of these colonies were analyzed for pathogen prevalence and abundance (n = 28), of which two died before November 2014, and were replaced by three new colonies bringing the total unique colonies described in this dataset to 31. Each beekeeping operation administered anti-mite treatments. To reduce *Varroa destructor* mite infestation levels in 2014, Operation 1 treated colonies with amitraz on April 22 and Sept. 4, and oxalic acid on Oct. 20, and Operation 2 treated colonies with amitraz on March 15, oxalic acid on Aug. 21, amitraz on Sept. 9, and formic acid on Oct. 24. Colony health, using colony population size as a proxy for health, was estimated by counting and imaging the number of frames more than two-thirds covered with bees. Colony health was categorized as weak (< 7 frames), average (7–12 frames), or strong (> 12 frames) based on frame count (S3 Table) [20,21]. Samples of honey bees were obtained from monitored colonies, up to four times each. Specific 2014 sample dates varied and were categorized into discrete sampling events in order to better compare data within and between colonies managed by two beekeeping operations. Specifically, “before almond pollination” samples were obtained from colonies located in California in January 2014 (Beekeeping Operation 1 on Jan. 27 and Beekeeping Operation 2 on Jan. 29). Samples that represent the pathogens associated with honey bee colonies “during almond pollination” were obtained immediately post-almond pollination in March 2014 (Beekeeping Operation 1 on March 3 and Beekeeping Operation 2 on March 14). “After almond pollination” samples were obtained from colonies that were located in Minnesota in early (June 9) and late (Aug. 25 and Sept. 2) summer (Fig 1 and S3 Table). Additionally, core samples containing pollen/bee bread, wax, and honey were obtained from a representative frame in each colony and analyzed for pesticide residues, as described in Kegley et al. 2017 [50]. The sample cohort analyzed herein includes pathogen prevalence, abundance, and survivorship data obtained in 2014, including variable numbers of samples obtained from dead (n = 2), weak (n = 8), average (n = 34), and strong (n = 48) rated colonies (total = 92) spanning the duration of the 2014 California almond pollination season, and colony longevity data through January 2015 (Fig 1 and S3 Table). In total, 21 colonies in this sample cohort died by the end of January 2015.

### Honey bee samples

At each sampling event, live honey bees (~ 150 per sample) were obtained from a frame containing developing bees (brood) in the center each colony. Samples were composed of female bees of mixed age. Collected samples were placed on ice or dry ice in the field, stored at -20°C, transported on dry ice, and transferred to -80°C for storage prior to analysis. Five female bees from each sample were used for RNA extraction, cDNA synthesis, pathogen-specific PCR, and qPCR. The objective for pathogen screening was to identify the most prevalent pathogens associated with honey bees sampled from individual colonies at each sampling event. Based on empirical data, literature values, and practical sample handling considerations, we assayed five bees per colony per sampling event [22]. The following equation from Pirk et al. 2013, N = ln(1-D) / ln(1-P) (N = sample size, ln = natural logarithm, D = probability of detection, P = proportion of infected bees) predicts that with a sample size of five bees, pathogenic infections affecting 45% or more of the individuals within a colony would be detected with 95% probability [51]; this sample size has proven sufficient for the pathogen-specific PCR detection of highly prevalent pathogens [21–23].

### *Varroa destructor* mite infestation level

*Varroa destructor* mite infestation levels for each colony were estimated using the alcohol wash technique [52]. At each sampling event, 150 adult bees were obtained from a brood-containing frame and rinsed in ethanol to dislodge attached mites into a collection jar. Mites infestation percentage was reported as the number of mites per 100 bees [52]. In total 92, samples were included in the analysis (Fig 1 and S3 Table).

### RNA isolation

Five female bees per sample were homogenized together in water using beads (3 mm) and a TissueLyzer (Qiagen) at 30 Hz for 2 minutes. Samples were centrifuged for 12 minutes at 12,000 x g at 4°C to pellet debris, and RNA from supernatants was extracted using Trizol reagent (Life Technologies) according to the manufacturer’s instructions [21–23].

### Reverse transcription/cDNA synthesis

cDNA synthesis reactions were performed by incubating 2 μg of total RNA extracted from honey bee samples, Moloney murine leukemia virus (M-MLV) reverse transcriptase (Promega), and 500 ng random hexamer primers (Integrated DNA Technologies) for 1 hour at 37°C, according to the manufacturer’s instructions [21,22].

### Polymerase Chain Reaction (PCR)

Pathogen specific PCR was performed according to standard methods using the primers listed in S1 Table. Specifically, PCR was used to determine the prevalence of DWV, BQCV, IAPV, KBV, LSV1, LSV2, *N*. *ceranae*, and *L*. *passim* (S1 Table). In brief, each PCR included 1 μl cDNA template, combined with 10 pmol each of forward and reverse pathogen specific primers, and amplified with ChoiceTaq polymerase (Denville) according to the manufacturer’s instructions using the following cycling conditions: 95°C for 5 minutes; 35 cycles of 95°C for 30 seconds, 55°C for 30 seconds, and 72°C for 30 seconds, followed by final elongation at 72°C for 4 minutes. The PCR products were visualized using 2% agarose gel electrophoresis followed by fluorescence imaging. In addition, the PCR assays utilized in this study were verified by sequencing [22]. Positive and negative control reactions were included for all pathogen-specific PCR analyses and exhibited the expected results. IAPV was detected by PCR in only one of the samples, thus IAPV abundance was not examined by quantitative PCR.

### Quantitative Polymerase Chain Reaction (qPCR)

Quantitative PCR was used to determine the relative pathogen abundance in each sample. Specifically, qPCR was used to determine the abundance of DWV, BQCV, IAPV, KBV, LSV1, LSV2, *N*. *ceranae*, and *L*. *passim* (S1 Table). All qPCR reactions were performed in triplicate with a CFX Connect Real Time instrument (BioRad) and the following reaction conditions: 2 μL of cDNA template in 20 μL reactions containing 1X ChoiceTaq Mastermix (Denville), 0.4 μM each forward and reverse primer, 1X SYBR Green (Life Technologies), and 3 mM MgCl2. The qPCR thermo-profile consisted of a single pre-incubation 95°C (1 minute), 40 cycles of 95°C (10 seconds), 58°C (20 seconds), and 72°C (15 seconds). Plasmid standards, containing from 10^9^ to 10^3^ copies per reaction, were used as qPCR templates to assess primer efficiency and quantify the relative abundance of each pathogen. The linear standard equation for primer efficiency of each primer set, which was generated by plotting the crossing point (Cp) versus the log_10_ of the initial plasmid copy number, is as follows: BQCV: y = -3.7336x+ 42.849, R^2^ = 0.996; DWV: y = -3.443x + 41.277, R^2^ = 0.99958; LSV1: y = -3.1994x + 38.71, R^2^ = 0.982, LSV2: y = -3.8147x + 44.805, R^2^ = 0.980; KBV: y = -3.4505x + 40.099, R^2 = 0.99927, *L*. *passim*: y = -3.4825x + 41.025, R^2^ = 0.98588 and *N*. *ceranae*: y = -3.9656 + 42.124, R^2^ = 0.9961; the minimum qPCR detection levels were ≤ 1,000 copies per sample. In addition, qPCR of a host encoded gene, *Apis m*. Rpl8, was performed using 2 μL cDNA template on each qPCR plate to ensure consistency and cDNA quality. qPCR products were analyzed by melting point analysis and 2% agarose gel electrophoresis. In addition, the qPCR assays utilized in this study were verified by sequencing [22]. An estimate of the number of viral RNA copies per bee can be obtained by multiplying the reported qPCR copy number values by 25. This estimate is based on the following: typical RNA yield was approximately 50 μg per bee, each qPCR reaction was performed on cDNA generated from 2 μg RNA, therefore each well represents 1/25th of an individual bee [21].

### Statistical analysis of PCR data

For this study, we use “pathogen prevalence” to refer to the sum of the number of pathogens detected by PCR out of seven total; an additional pathogen was counted (+1) when *Varroa* mite infestation surpassed 3%. A one-way ANOVA was used to compare pathogen prevalence between colonies with differential colony health ratings as they were graded at the time of sampling (i.e., dead, strong, average, and weak), and to compare pathogen prevalence between sampling events (i.e., before, during, after 1, after 2, and after 3 almond pollination).

### Statistical analysis of qPCR data with multiple linear regression

The associations between pathogen abundance and other monitored factors, including colony strength, mite count, and beekeeping operation were evaluated for each pathogen using multiple linear regression [22]. The natural logarithm (ln) was used to transform pathogen abundance (as determined by qPCR) to normalize the response variable; 1 was added to each observation since some observations had 0 total abundance. The confounding effects of seasonality on pathogen abundance was removed by using the residuals of a linear regression evaluating the relationship between pathogen abundance and the “day of year” of sampling as the response variable in the multiple linear regression models. As a result, models reported associations between the residuals of pathogen abundance after accounting for the effects of pathogen seasonality (“residual abundance”), and colony strength rating, beekeeping operation, and mite count. Most colonies were measured multiple times in the 92 samples; therefore, a random effect for individual colony was included in the models to account for repeated measures. Estimated models for each pathogen are described as:

$$\text{ln}\left({\text{y}}_{\text{i}}\right){= \beta }_{\text{0}}{+ \beta }_{\text{1}}{\text{x Operation 2}}_{\text{i}}{+ \beta }_{\text{2}}{\text{x Frame Count}}_{\text{i}}{+ \beta }_{\text{3}}{\text{x Mites}}_{\text{i}}{+ \gamma }_{\text{ij}}{+ \varepsilon }_{\text{i}}$$

Where ln(y_i_) represents the residuals of pathogen abundance in response to day of year of sampling for each pathogen, β_0_ is the estimate of the model intercept, β_1_ is the estimate of the change in pathogen abundance of Operation 2 compared to Operation 1, β_2_ is the estimate of the change in pathogen abundance for each unit increase in frame count, and β_3_ is the estimate of the change in pathogen abundance for each unit increase in mite count. The variable γ_i(j)_ represents a random effect for colony *i* sampled *j* times, and ε_i_ represents the residual error from the model. Coefficients (est) of the estimated linear model describe the magnitude and direction of associations between the residual abundance of individual pathogens (i.e., DWV, BQCV, KBV, LSV1, LSV2, *L*. *passim*, and *N*. *ceranae*) and other monitored factors throughout 2014.

### Statistical analysis of *Varroa destructor* mite infestation with binomial regression

A generalized linear mixed effects model with a binomial family distribution and random effect for individual colony was used to estimate the odds that a colony will surpass the threshold of 3% mite infestation, after which treatment is recommended, in response to the date of sampling. Colonies were assigned a value of “1” when mite infestation surpassed 3%, and were assigned a value of “0” when *Varroa* mite infestation was less than 3%. The odds that a colony would surpass the 3% mite infestation threshold were plotted in response to the day of year and a best-fit line was fit to the data.

### Multidimensional statistical analysis of pathogen compositions

We defined the pathogen composition of a honey bee colony as the abundance of seven pathogens (DWV, BQCV, LSV1, LSV2, KBV, *L*. *passim*, and *N*. *ceranae*) relative to all other samples. We used the Bray-Curtis index to create a dissimilarity matrix using the natural logarithm (ln) of pathogen abundance of seven pathogens. The relative composition of pathogens was visualized using nonmetric Multidimensional Scaling (NMDS). Relative differences in colony pathogen compositions were visualized using the five discrete categories of sampling events; January (California, before), March (California, during), June (Minnesota, after 1), August (Minnesota, after 2), and September (Minnesota, after 3).

We used a permutational analysis of variance (PERMANOVA) to test the ability of sampling event, mite count, and colony health to explain variation within the NMDS plot [53]. The main effects as well as an interaction between sampling event and mite count were included in the reported PERMANOVA model. Colony health rating was included as a main effect in a separate PERMANOVA model, but was not considered a significant predictor. Additionally, we described any differences in the variability of pathogen composition by sampling event using an analysis of multivariate homogeneity of group dispersions (betadisper) [53]. Furthermore, we used a similarity percentage (SIMPER) analysis to describe the percent dissimilarity explained by each pathogen in comparisons between consecutive sampling events [53].

### Statistical software

All statistical analysis was performed using the software “R” (R version 3.1.3, “Smooth Sidewalk”) [54]. Generalized linear models were conducted using the nlme package [55]. NMDS plots were generated using the labdsv package while all other multivariate analyses were performed using the vegan and labdsv packages [53,56].

## Results and discussion

### Honey bee colony monitoring and pathogen diagnostics

Commercially managed honey bee colonies that were transported from Minnesota to California for almond pollination were monitored from January 2014 to January 2015 (Fig 1) [50]. At each sampling event, colony health, using colony population size as a proxy for health, was monitored and samples of live honey bees were obtained for pathogen diagnostics, including *Varroa destructor* mite infestation levels and the prevalence and abundance of five commonly occurring viruses (i.e., BQCV, DWV, LSV1, LSV2, and KBV), and two eukaryotic pathogens (the trypanosomatid *Lotmaria passim* and the microsporidial pathogen *Nosema ceranae*) using pathogen specific PCR and qPCR, respectively [22,23,26,50]. Israeli acute paralysis virus (IAPV) was only detected in one sample using PCR, thus it was not included in further analysis. In this work, we use “pathogen prevalence” to indicate the number of different pathogens detected in a sample and “pathogen abundance” as the estimated number of relative RNA transcripts for each pathogen using RT-qPCR (S3 Table). Furthermore, we use “pathogen composition” to refer to the abundance of all co-infecting pathogens in a sample relative to all other samples. Therefore, both pathogen prevalence and abundance affect the pathogen composition of each colony throughout the course of the study.

### Pathogen prevalence and colony health

To examine the relationship between pathogen prevalence and colony health, the total number of pathogens detected in each sample, using PCR results for seven pathogens (i.e. DWV, BQCV, LSV1, LSV2, KBV, *L*. *passim*, and *N*. *ceranae*), and *V*. *destructor* mite infestation when levels were above the recommended treatment threshold of 3% (i.e., 3 mites per 100 bees) was averaged by colony health rating (i.e., weak, average, strong, or dead) (S3 Table) [7,52,57,58]. Mean pathogen prevalence varied by colony health rating (ANOVA, *F*_*3*,*88*_ = 3.05, *p*-value = 0.03). Specifically, samples from dead colonies averaged 6.50 pathogens (n = 2, SE +/- 1.50), weak colonies 5.25 pathogens (n = 8, SE +/- 0.38), average colonies 5.38 pathogens (n = 34 SE +/- 0.18), and strong colonies 5.95 pathogens (n = 48, SE +/- 0.13) (S2 Table and S1 Fig). Strong rated colonies had higher pathogen prevalence than average rated colonies (S1 Fig). No other pairwise comparisons of mean pathogen prevalence between colony health ratings were statistically different.

The mean pathogen prevalence associated with each colony varied with the sampling date (ANOVA, *F*_*4*,*87*_ = 21.31, *p*-value < 0.001) (S3 and S4 Tables and S2 Fig). Specifically, colonies sampled early in the year before almond pollination averaged 5.00 pathogens (n = 28, SE +/- 0.17), and colonies sampled in March, immediately after almond pollination and thus representing the pathogen profile of colonies during the almond bloom, averaged 5.39 pathogens (n = 28, SE +/- 0.09). Colonies sampled in early summer when the colonies were in Minnesota (i.e., after 1, June) averaged 7.09 pathogens (n = 11, SE +/- 0.28), in late summer (i.e., after 2, Aug.) colonies averaged 6.43 pathogens (n = 14, SE +/- 0.13), and at the end of summer (i.e., after 3, Sept.) colonies averaged 6.00 pathogens (n = 11, SE +/- 0.23) (S4 Table and S2 Fig). Results from Tukey’s HSD *post-hoc* tests indicate that the mean pathogen prevalence was greater in sampling events later in the year, when the colonies were located in Minnesota (after 1, 2, and 3) compared to sampling events in January and March, before and during almond pollination, respectively, when the colonies were located in California (S2 Fig).

### Longitudinal monitoring of pathogen composition

**Fig 2:**
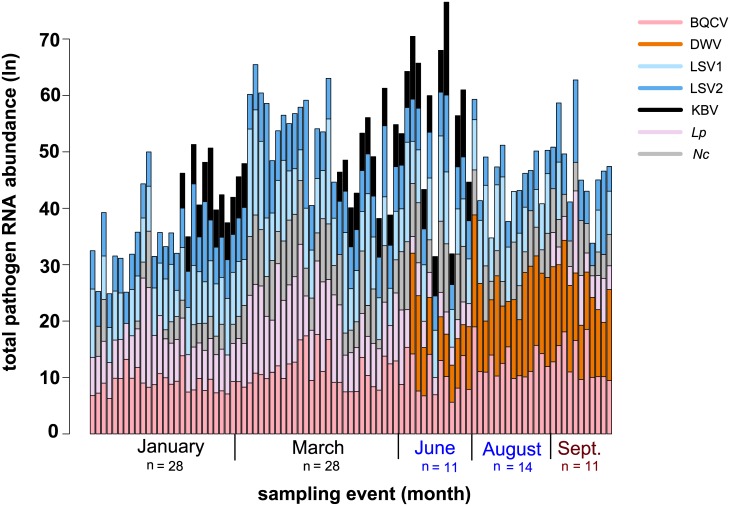
Longitudinal assessment of honey bee pathogen abundance at the colony level. Spatiotemporal change of the pathogen composition of individual honey bee colonies is designated by each vertical bar. In this sample cohort, the most prominent changes are attributed to the detection of DWV and reduced abundance of *L*. *passim* (*Lp*) in June, August, and September. Quantitative PCR was used to determine the relative pathogen abundance (S3 Table). The natural log transformed data for each pathogen, BQCV (pink), DWV (orange), *Lp* (light pink), *Nc* (gray), LSV1 (light blue), LSV2 (dark blue), and KBV (black), is represented by the height of each segment of the vertical bar representing a sample from an individual colony (y-axis); the height of the bar represents the total pathogen abundance for each colony over the course of the study (January to September 2014, x-axis).

To examine the relationships between pathogen abundance and other monitored factors (i.e., colony health, sample date, percent mite infestation, and beekeeping operation) we performed mite counts at each sampling event and utilized qPCR to quantify the relative abundance of seven pathogens (i.e. DWV, BQCV, LSV1, LSV2, KBV, *L*. *passim*, and *N*. *ceranae*) (S3 Table). Quantitative PCR values ranged from 0 to 6.7x10^8^ per sample (S3 Table). Overall, longitudinal assessment of the abundance of common honey bee pathogens at the colony level from January to September 2014 revealed that individual colonies, both within a single beekeeping operation and between two typically managed commercial beekeeping operations, varied from the beginning of the monitoring period in January, until the end of the monitoring period in September (Fig 2 and S3–S9 Figs).

**Fig 3:**
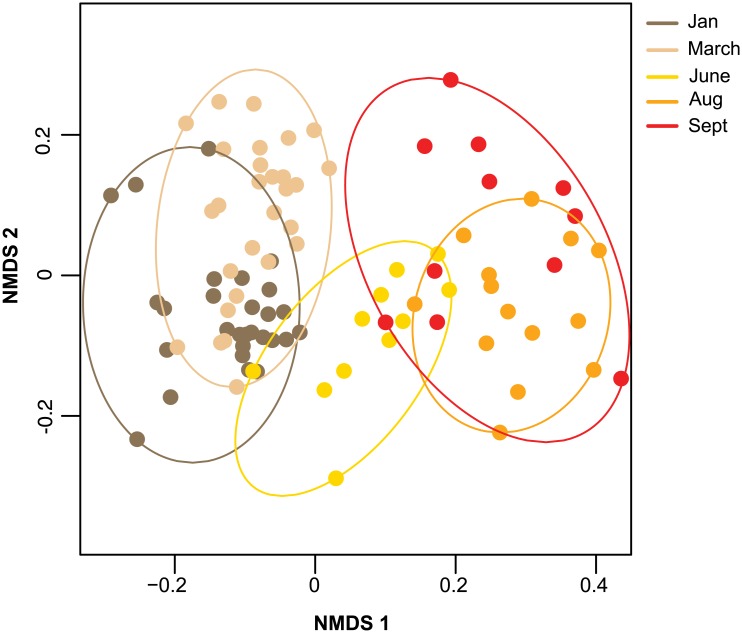
Relative pathogen composition of honey bee colonies visualized by sample event. Pathogen compositions of honey bee colonies form unique and defined clusters according to the month they were sampled (i.e., January—dark brown, March—light brown, June—yellow, August—orange, or September—red). The position of each point indicates the pathogen composition of each sample relative to all other samples (i.e. samples with more similar pathogen compositions are closer), calculated using a Bray-Curtis dissimilarity and plotted on a non-metric multidimensional scaling (NMDS) plot with an associated stress value of (0.197); the results from a permutational analysis of variance (PERMANOVA) in Table 1 indicated that the sampling event explained the most amount of variance in the pathogen composition.

To investigate if weak and strong colonies had different pathogen compositions, we quantified the differences in pathogen prevalence and abundances between honey bee samples using a Bray-Curtis dissimilarity matrix. The pathogen composition of each sample relative to all other samples were visualized using a non-metric multidimensional scaling (NMDS) plot (Fig 3). Results from a PERMANOVA analysis indicated that sampling date explained the most variation in pathogen compositions (Fig 3 and Table 1, R^2^ = 0.61, *p <* 0.01), followed by levels of *Varroa destructor* mite infestation (Table 1, R^2^ = 0.01, *p =* 0.05), indicating pathogen composition varies seasonally and with changing levels of mite abundance [53]. Unexpectedly, pathogen composition did not differ between colony health ratings (PERMANOVA, *F*_*3*,*83*_ = 1.467, *p* = 0.14, R^2^ = 0.018). To identify the specific pathogens that contributed the most to the changes in pathogen composition between sampling events, a SIMPER analysis was used to calculate the contribution of each pathogen to the dissimilarity of pathogen compositions between consecutive sampling events (Table 2) [53]. Differences in pathogen composition of honey bee colonies between consecutive sampling events was not consistently explained by a single pathogen, but varied for each comparison, highlighting the dynamic nature of honey bee colony pathogen composition. DWV contributed the most to the differences between pathogen compositions of honey bee colonies when the mean difference in *V*. *destructor* mite infestation was greatest (Table 3), likely due to the role of mites in DWV transmission. Results from a multivariate homogeneity of group dispersions analysis (betadisper) indicated that the dispersion of pathogen composition among sampling events did not change (F_4,87_ = 0.27, *p*-value = 0.90) [53]. Together, this analysis indicated that the composition of honey bee associated pathogens is most influenced by sampling date and the level of mite infestation.

**Table 1:** Analysis of variance of pathogen composition. The relative pathogen composition of honey bee samples, based on all pathogen abundances, is primarily explained by the date the sample was obtained (or “day of year”) and level of *Varroa* mite infestation. Results from a permutational analysis of variance (PERMANOVA) indicate that the day of year (R^2^ = 0.61), followed by the percent mite infestation (R^2^ = 0.01) explained the most variation in the relative pathogen composition of all honey bee samples; factors contributing significantly to the relative pathogen composition are indicated by *p*-values ≤0.05 in bold.

Source	d.f.	*F*-Statistic	R^2^	*p*-value
*day of year*	4	34.93	0.61	**<0.01**
*mite infestation (%)*	1	2.69	0.01	**0.05**
*sampling event* x *mite infestation (%)*	4	0.81	0.01	0.63
*residuals*	83	-	0.36	
*Total*	92	-	1.00	

**Table 2:** Similarity percentage analysis of the relative pathogen composition between consecutive sampling events. The greatest differences in honey bee colony pathogen composition was explained by changes in abundance of a different pathogen between consecutive sampling events. The percent difference attributed to each pathogen between consecutive sampling events was calculated using a similarity percentage (SIMPER) analysis of a Bray-Curtis dissimilarity matrix. The cumulative difference of the top three pathogens in comparisons between consecutive sampling events is reported as a percentage. The difference in percent mite infestation was calculated in comparisons between consecutive sampling events. When the change in mean mite infestation was greatest, DWV accounted for the most difference in honey bee colony pathogen composition.

*Sampling Event*	*Pathogen1*	*Pathogen 2*	*Pathogen 3*	*Cumulative Dissimilarity (%)*	*Δ mean mite infestation (%)*
*Jan-Mar*	*Nc*	*Lp*	LSV1	60.0	0.02
*Mar-Jun*	DWV	*Lp*	KBV	58.0	6.19
*Jun-Aug*	KBV	DWV	*Nc*	57.0	3.01
*Aug-Sept*	LSV1	*Lp*	LSV2	63.0	-5.09

**Table 3:** Pathogen abundance model summaries. Multiple linear regression models were utilized to identify associations between colony-level residual pathogen abundance and the factors monitored in this study (i.e., colony health, percent mite infestation, and beekeeping operation) throughout the monitoring period from January to September 2014. Residual pathogen abundance was used as the response variable in the models to remove the confounding effect of seasonality on pathogen abundance in order to investigate associations between residual pathogen abundance and other covariates. Associations between the residual abundance of each pathogen assayed in this sample cohort and other covariates are denoted by the magnitude and direction of the estimate listed in the table. Operation 2 estimates the residual pathogen abundance in comparison to Operation 1, frame count was used as a continuous variable as an estimate of colony health, and mite infestation (%) represents the number of mites counted per 100 bees. The standard error, t-value, and p-value for each estimate of coefficients (est.) within the final model are provided; *p*-values <0.05 are indicative of significant associations (bold).

***BQCV***	***estimate***	***SE (+/-)***	***t-value***	***p-value***
*intercept*	0.47	0.93	0.50	0.612
*operation 2*	-1.51	0.68	-2.23	**0.03**
*frame count*	0.01	0.07	0.16	0.87
*mite infestation (%)*	0.06	0.06	1.06	0.29
***DWV***	***estimate***	***SE (+/-)***	***t-value***	***p-value***
*intercept*	0.42	0.77	0.55	0.59
*operation 2*	-0.37	0.52	-0.71	0.48
*frame count*	-0.09	0.06	-1.49	0.15
*mite infestation (%)*	0.23	0.05	4.84	**<0.001**
***LSV1***	***estimate***	***SE (+/-)***	***t-value***	***p-value***
*intercept*	-1.52	1.38	-1.10	0.27
*operation 2*	2.27	0.95	2.38	**0.02**
*frame count*	0.03	0.10	-0.33	0.74
*mite infestation (%)*	-0.02	0.08	-0.23	0.82
***LSV2***	***estimate***	***SE (+/-)***	***t-value***	***p-value***
*intercept*	-0.54	0.98	-0.54	0.59
*operation 2*	-0.41	0.68	-0.61	0.55
*frame count*	0.09	0.08	1.19	0.24
*mite infestation (%)*	-0.07	0.06	-1.14	0.26
***KBV***	***estimate***	***SE (+/-)***	***t-value***	***p-value***
*intercept*	-1.59	0.82	1.94	0.06
*operation 2*	5.73	0.56	-1.95	**<0.001**
*frame count*	-0.11	0.06	-1.74	0.09
*mite infestation (%)*	-0.09	0.05	-1.91	0.06
***L*. *passim***	***estimate***	***SE (+/-)***	***t-value***	***p-value***
*intercept*	-0.53	1.02	-0.52	0.61
*operation 2*	-3.54	0.70	-5.07	**<0.001**
*frame count*	0.23	0.08	2.94	**0.005**
*mite infestation (%)*	-0.01	0.06	-0.19	0.84
***N*. *ceranae***	***estimate***	***SE (+/-)***	***t-value***	***p-value***
*intercept*	-1.92	1.22	-1.57	0.12
*operation 2*	0.20	0.83	0.24	0.81
*frame count*	0.18	0.09	1.99	**0.05**
*mite infestation (%)*	-0.06	0.07	-0.75	0.46
***total abundance***	***estimate***	***SE (+/-)***	***t-value***	***p-value***
*intercept*	-0.33	1.02	-0.33	0.75
*operation 2*	-1.49	0.73	-2.05	**0.05**
*frame count*	0.08	0.08	1.00	0.32
*mite infestation (%)*	0.10	0.06	1.64	0.11
***odds surpassing Varroa threshold***	***estimate***	***SE (+/-)***	***t-value***	***p-value***
*Intercept*	-51.02	14.35	-3.56	**<0.001**
*day of year*	0.26	0.07	3.66	**<0.001**

### *Varroa destructor* and colony health

*V*. *destructor* mites are a major contributor to honey bee colony losses [10,25,44,57,59].

**Fig 4:**
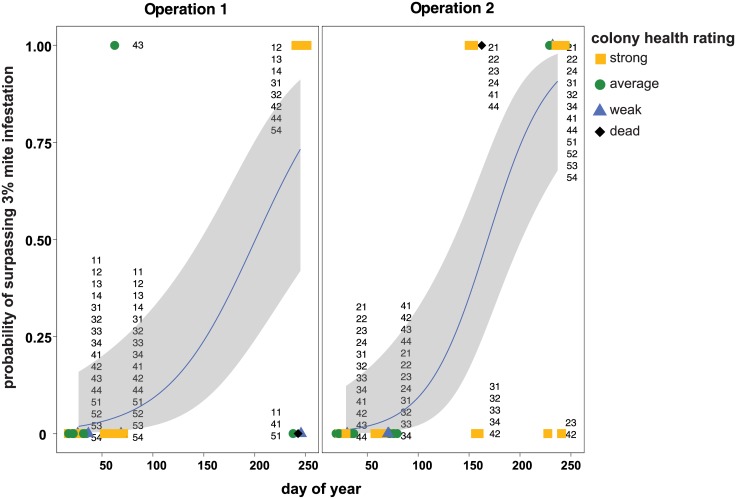
The probability of honey bee colonies accumulating *Varroa destructor* mite densities above the threshold recommended for treatment is greater later in the year. Honey bee colonies have a high probability of accumulating levels of *Varroa destructor* mites that surpass the threshold for recommended treatment (i.e., 3%) by the end of the pathogen monitoring period in both beekeeping operations. Results from a generalized linear mixed effects model with a binomial family distribution indicate that by the end of the sampling period, colonies had a 99.0% chance of crossing the recommended treatment threshold. Since mite count data differed between beekeeping operations, binomial regression results were plotted independently. Mite count data obtained from honey bee samples collected from colonies rated dead (black diamonds), weak (blue triangles), average (green circle), and strong (yellow square) are shown with their unique colony identifier numbers, with the first digit identifying the pallet and the second digit identifying individual colony. A best-fit line (blue) with odds-estimates surrounded by upper and lower standard error estimates (gray) depicts the odds of a colony surpassing the recommended treatment threshold (y-axis), which increases with the day of the year (x-axis).

*V*. *destructor* mite infestation increased over the course of this study. Specifically, honey bee samples from colonies early in the year, when the colonies were located in California, harbored a mean mite infestation of 0.45% (mean = 0.45, SE +/- 0.09, n = 56) (S3 Table). Whereas, honey bee samples obtained from colonies later in the year, when the colonies were located in Minnesota after almond pollination (i.e. after1, after2, and after3), harbored a 7.17% mean mite infestation (mean = 7.17, SE+/- 1.21, n = 36) (S3 Table). To evaluate the risk *Varroa* mites pose to colonies throughout the sampling period, results from a generalized linear mixed effects model with a binomial family distribution demonstrate that the odds of a colony exceeding the recommended treatment threshold level of 3% *Varroa* mite infestation is e^0.26^ (1.3) times higher with each increase in the day of the year (log-odds of surpassing threshold, est = 0.23) (Fig 4 and Table 3). These results indicate that honey bee colonies within this sample cohort frequently accumulated higher levels of mites toward the end of the pathogen monitoring period (Fig 4), consistent with the typical mite population growth pattern that tracks the seasonal colony buildup and decline over the course of a year.

In total, 21 colonies in this sample cohort died by the end of the sampling period in January 2015. From January 2014 to January 2015, 22 of the monitored colonies surpassed the 3% mite infestation threshold, and of those colonies that died, 40.9% (9/22) of colonies surpassed the 3% mite infestation threshold by the end of the sampling period. However, the percentage of colonies that had at some point in the study surpassed the 3% mite infestation threshold and died differed by beekeeping operation; Beekeeping Operation 1 experienced 22.2% loss (i.e., 2 of 9 colonies with > 3% mite infestation) and Beekeeping Operation 2 experienced 53.8% loss (i.e., 7 of 13 colonies with > 3% mite infestation) (S3 Table). It is notable that both beekeeping operations had numerous colonies with high levels of mite infestation, despite application of several anti-mite treatments including amitraz, oxalic acid, and formic acid over the course of the sampling period (see Methods).

### Association of pathogen abundance and monitored factors, after accounting for sample date

Individual pathogen abundance was consistently affected by the sample date (“day of year”) (S3–S9 Figs), therefore we further examined the relationship between pathogen abundance and other factors (e.g., beekeeping operation, colony health, and mite infestation) after removing the confounding effects of sample date. In order to remove the confounding effects of sample date on pathogen abundance, we examined the relationship between the residuals of a linear regression of pathogen abundance in response to “day of year” (i.e., residual abundance), and used residual abundance as the response variable in multiple linear regression to evaluate associations with residual abundance and beekeeping operation, mite infestation percentage, and colony health, which was represented in the models as frame count (Table 3).

**Fig 5:**
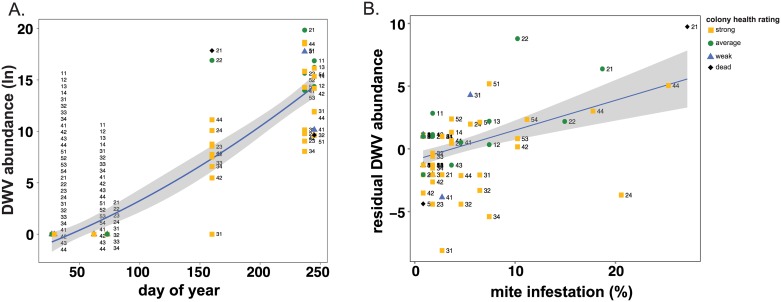
DWV abundance increased from January to September 2014. DWV abundance was greatest in honey bee samples with higher mite infestation levels, obtained later in the year (June through September). A. The natural log transformed values of the relative DWV RNA abundance as determined by qPCR (y-axis) analysis of honey bee samples obtained from colonies with varying colony health ratings (i.e., dead (black diamonds), weak (blue triangles), average (green circle), and strong (yellow square)); the date of sample collection (i.e. day of year, x-axis). The best-fit line (blue), surrounded by upper and lower standard error estimates (gray), indicates that DWV abundance increased at an exponential rate across the longitudinal monitoring period. Unique colony identifier numbers, with the first digit identifying the pallet and the second digit identifying individual colony, label each point on the graph and illustrate changes in the pathogen abundance individual colonies throughout the sampling period. B. The residual abundance of the natural log transformed values of DWV RNA abundance (y-axis) increases as the level of mite infestation (x-axis) increases.

Concurrent with increased *V*. *destructor* mite levels were increased DWV prevalence and abundance (Figs 2 and 5). All the honey bee samples in this sample cohort (n = 92) were tested for the presence of DWV using PCR (S3 Table). Interestingly, none of the samples collected in January and March when the colonies were located in California (n = 56) were DWV positive, whereas 96.4% of samples obtained from colonies located in Minnesota (n = 36) were positive for DWV. Quantitative PCR was utilized to determine the relative DWV abundance in each sample (n = 92), which in general increased over the course of the monitoring period (Fig 5 and S3 Table). Multiple linear regression was used to investigate associations between residual DWV abundance with the predictor of interest, frame count (i.e., colony strength), while also accounting for beekeeping operation and level of mite infestation, indicating that median residual DWV abundance increased by e^0.23^ (126%) for each unit increase in mite infestation after accounting for beekeeping operation and frame count (Table 3). These data support previous results describing the relationship between mite infestation and DWV abundance [7,14,45].

KBV was only detected in Operation 2, and as a result residual abundance was estimated in the model to be greater within Operation 2 when compared to Operation 1, while also accounting for frame count and mite infestation percentage (Table 3). While not significant, a negative association was observed between residual KBV abundance and levels of mite infestation (mites, est = -0.09, SE = 0.06, *p*-value = 0.06) (Table 3). Mites transmit both DWV and KBV, therefore the positive correlation between DWV and mite infestation was expected, whereas further investigation is required to determine the relationship between KBV and mite infestation [44,46,47].

The residual abundance patterns of other viruses did not consistently associate with the levels of *Varroa destructor* mite infestation. LSV2 is a member of the recently discovered and described Lake Sinai virus group, which is prevalent, abundant, and globally distributed [21–23,31,60,61]. Consistent with other studies, LSV2 abundance was higher in weak colonies, as compared to healthy colonies with greater bee populations in this sample cohort [23,28], but this observation was only statistically supported when colony health was categorized into health ratings based on frame count. The median residual LSV2 abundance was e^7.39^ (161970.60%) greater in “dead” colonies compared to “weak” rated colonies (t-test, t-value_56_ = 3.02, *p* = 0.004), when categorical colony health data, as opposed continuous frame count data, was used in the models, while also accounting for the effect of beekeeping operation and mite infestation. However, samples were only collected from 2 of the 22 colonies that died. Despite the close phylogenetic relationship of LSV1 and LSV2 [23], changes in LSV1 abundance were associated with monitored factors that were different from those that correlated with LSV2 abundance. Although LSV1 was similarly widespread, testing positive in 86.9% of samples (80/92) (S3 Table), median residual LSV1 abundance was e^2.27^ (967.94%) times greater in Operation 2 compared to Operation 1 when also accounting for frame count and mite infestation (Operation 2, est = 2.27, SE ± 0.95, *p* = 0.02) (Table 3) (S6 Fig).

Counterintuitively, the residual abundance of *L*. *passim* and *N*. *ceranae* increased with colony population size estimated by frame count (Table 3). The median residual abundance of *L*. *passim* increased by e^0.23^ (125%) with each unit increase in frames counted, and the median residual abundance of *N*. *ceranae* increased by e^0.18^ (119.72%) in response to each unit increase in frames counted when also accounting for the effects of beekeeping operation, and mites (Table 3). These results differ from previous studies demonstrating that *N*. *ceranae* influences co-infections of other pathogens important to reduced colony health in the US [10,28,49], or as the causative agent of colony loss in Spain [62,63].

Furthermore, we examined correlations between changes in individual colony health between time points (Δ-Frame Count) and pathogen abundance (S10 Fig). For all pathogens except KBV, there was no correlation between abundance and Δ-Frame Count independent of day of year (S10 Fig). There was a negative correlation between KBV abundance and Δ-Frame Count independent of day of year (S10 Fig).

### Pathogen abundance and colony health evaluated by beekeeping operation

The abundance of each pathogen associated with samples obtained from monitored honey bee colonies managed by two commercial beekeeping operations was plotted as function of time (i.e., day of the year) (S3–S9 Figs). Together these data illustrate the similarities and differences in pathogen loads over the course of this study, and between colonies managed by different beekeeping operations, which encompasses numerous confounding variables including precise geographic location, intensities of exposures to pathogens, honey bee colony genetics, the availability and quality of bee forage, and specific management practices, all of which may impact colony health and pathogen prevalence and abundance. In this sample cohort, the abundances of BQCV, LSV1, KBV, and *L*. *passim*, and the total pathogen abundance differed between beekeeping operations (Table 3). However, there was a large gap in data between March and August for each beekeeping operation, thus this study cannot attribute differences in pathogen abundance to apiary management. In previous US based honey bee monitoring studies, the specific beekeeping operation affected pathogen abundances [22]; however, this was not observed in colonies monitored as part of the German Bee Monitoring Project [25]. Additionally, in other studies, apiaries affected by CCD had more weak colonies with higher pathogen prevalence within closer proximity to other weak colonies with high pathogen prevalence [10]. Future investigations aimed at better understanding the mechanisms and dynamics of pathogen transmission between colonies within the same apiary (e.g., via mites, foraging/pollen, robbing, beekeeper mediated) are important to developing strategies to reduce inter-colony transmission.

### Pairwise pathogen interactions

**Fig 6:**
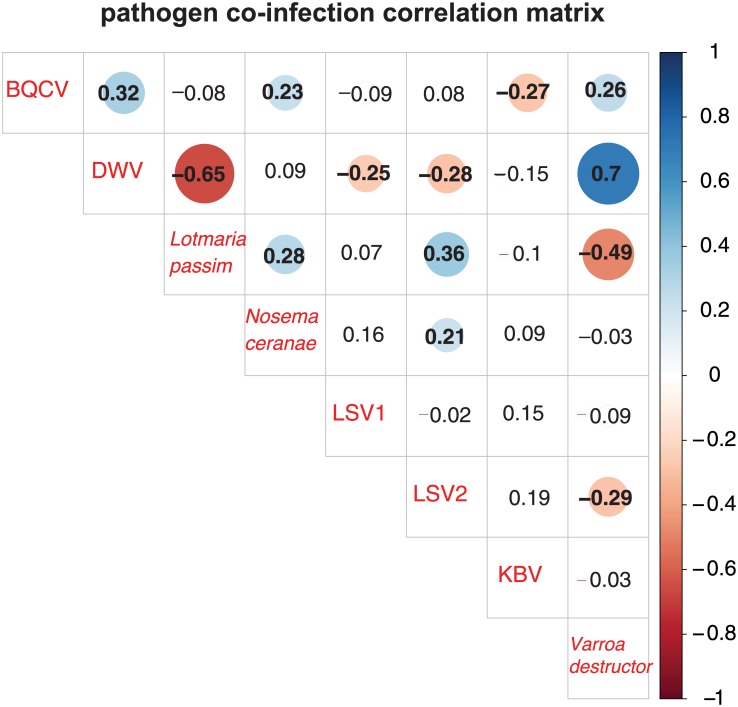
Pathogen co-infection correlation matrix. The abundance of pathogens in co-infected colonies was analyzed by calculating the correlation coefficients for each pair-wise comparison, which are listed in each cell. Correlation coefficients (reported as *r* values) quantify the strength and direction of the changes in pathogen abundance between pairs of pathogens. The shaded red circles represent negative correlations and blue circles represent positive correlations, darker hues and larger circles indicate stronger correlations, and bold numbers indicate significant correlations (*p*-value < 0.05). DWV and *Varroa destructor* mites had the strongest positive correlation, while DWV and *L*. *passim* displayed the strongest negative correlation.

Honey bee colonies are infected by a suite of pathogens that fluctuate in prevalence and abundance over the course of a year [10,22,28,31,64]. To investigate pairwise interactions between the pathogens monitored in this study, we generated a correlation matrix illustrating the correlation coefficients (R-values) that quantify the strength and direction of the relationship between pathogen abundance data (Fig 6). The strongest positive correlation observed in this sample cohort was between *V*. *destructor* mites and DWV (R = 0.70, *p*-value < 0.001). DWV and *L*. *passim* (R = -0.65, *p*-value < 0.001), followed by *V*. *destructor* mites and *L*. *passim* (R = -0.49, *p*-value < 0.001), demonstrated the strongest negative correlations (Fig 6).

## Conclusions

The health of commercially managed, migratory honey bee colonies in the US [7,10,21,22] and honey colonies located throughout the world [31,61,65–68] is influenced by multiple biotic and abiotic factors. In addition to pathogens, other factors including seasonal variation in colony size, foraging requirements, nutrition, and pesticide exposure all impact colony health [10,35,38,69,70]. Therefore, temporal monitoring projects that emphasize robust sampling designs (e.g., consistent monthly samples obtained on a specific date) and include large sample sizes and multiple monitored factors, are required to fully describe both general and acute threats to honey bee colonies throughout the year and in the context of pollination of specific crops. Additionally, the synergistic effects of co-infecting honey bee pathogens [35,64], which can impact the prevalence and abundance of other pathogens [7,28,31,49], must be investigated from a multidimensional approach to study the effects of pathogens on honey bee colony health. The combination of sampling individual honey bee colonies on specific days of the year and across multiple sites, and employment of statistical methods aimed at integrating and interpreting multifactorial data sets, will further our understanding of the factors with the greatest influence on colony losses and may result in the development of strategies to limit future losses.

## Supporting information

S1 Table Primers used in this study. (PDF)

S2 Table Pathogen prevalence by colony health rating. Pathogen prevalence varied significantly between strong and average colony health ratings. Honey bee samples obtained from dead, weak, average, and strong colonies were tested for the presence of seven pathogens, including five viruses (i.e., DWV, BQCV, LSV1, LSV2, KBV), *L*. *passim*, and *N*. *ceranae* using PCR, and for *Varroa destructor* mites, which were counted as a pathogen when infestation levels were above the recommended treatment threshold of 3%. Total pathogen prevalence was determined by summing the number of pathogens detected in each sample. Honey bee colony population size was used as a proxy for colony health by counting the number of frames more than 2/3 covered with bees (i.e. weak < 7 frames, average = 7–12 frames, strong >12 frames covered with bees). Included in the table are the mean number of pathogens per colony strength rating, the standard error estimate of the mean, and the number of colonies per colony health rating within this cohort. This sample cohort was comprised of 52.2% strong, 37% average, 8.6% weak, and 2.2% dead colonies.
(PDF)

S3 Table Complete data set. Honey bee colonies from two Minnesota-based commercial beekeeping operations that transport honey bee colonies to California for almond pollination were monitored for two years from January 2014 to January 2015. At the onset of the study in January 2014, beekeepers identified 12–16 queen-right colonies that successfully overwintered (n = 28), of which two died throughout the course of the study and were replaced by three novel colonies bringing the total unique colonies up to 31 throughout the course of the study (n = 31); repeated sampling of colonies resulted in 93 total samples. Honey bee colony population size was used as a proxy for colony health by counting the number of frames more than 2/3 covered with bees (i.e. weak < 7 frames, average = 7–12 frames, strong >12 frames covered with bees). Pathogen diagnostics was performed by PCR (1 = positive detection, 0 = not detected, NA—not assessed or no sample) and quantitative PCR (qPCR). Relative RNA equivalents were determined by qPCR and were natural log transformed for statistical analyses.
(XLS)

S4 Table Pathogen prevalence by sampling event. Pathogen prevalence varied significantly by sampling event. Honey bee samples were obtained from two Minnesota based, commercially managed honey bee colonies, twice when colonies were located in California before and immediately after almond pollination and thus reflect the pathogen prevalence during the almond bloom, and three times after the almond pollination event when colonies were located in Minnesota. Pathogen prevalence was measured by totaling the number of pathogens detected via PCR in each sample. Included are the average number of pathogens per sampling event, the standard error estimate of the mean, and the number of colonies per colony health rating within this cohort.
(PDF)

S1 Fig Analysis of mean pathogen prevalence by colony health rating. Mean pathogen prevalence is higher in samples from strong rated colonies, than in samples from average rated colonies. The number of pathogens detected by PCR, and mites (when infestation percentage surpassed 3 mites per 100 bees) were summed for each sample and averaged (y-axis) for each colony health rating (x-axis). Colony health was rated using colony size as a proxy, where colonies with <7 frames covered with bees were rated weak (n = 8, blue), 7–12 frames were rated average (n = 34), >12 frames were rated strong (n = 48, gold), or 0 frames were rated dead (n = 2, black). Points represent raw data, and center bars represent the mean colony pathogen prevalence, which are surrounded by the upper and lower standard error estimates. Significant differences between mean pathogen prevalence of colony health ratings was calculated using Tukey’s HSD *post-hoc* test (*p*-value < 0.05).
(EPS)

S2 Fig Analysis of mean pathogen prevalence by sampling event. Colonies acquired the highest mean number of pathogens by the first sampling event after almond pollination. The number of pathogens detected by PCR, and mites (when infestation percentage surpassed 3 mites per 100 bees) were summed in each sample and averaged (y-axis) for each sampling event (x-axis). Sampling events were conducted once before (n = 28, dark brown), once during (n = 28, light brown), and three times after California almond pollination (n = 11 yellow, n = 14 orange, and n = 11 red). Points represent raw data, and center bars represent the mean pathogen prevalence, which are surrounded by the upper and lower standard error estimates. Significant differences between the mean colony-level pathogen prevalence between sampling events were calculated using Tukey’s HSD *post-hoc* test (*p*-value < 0.05). The mean pathogen prevalence was greater in sampling events carried out after almond pollination when the colonies were located in Minnesota (after 1, 2, and 3) compared to sampling events before and during almond pollination when the colonies were located in California (with the exception of the pairwise comparison between during and after 3).
(EPS)

S3 Fig Changes in total pathogen abundance in honey bee colonies throughout the sampling period. Total pathogen abundance changed throughout the monitoring period, and was different between beekeepers. The natural log transformed values of individual pathogens measured by qPCR were summed (y-axis) and plotted by the date of sample collection (i.e. day of year, x-axis). Live honey bee samples were collected from colonies rated dead (black diamonds), weak (blue triangles), average (green circle), and strong (yellow square)). Unique colony identifier numbers, with the first digit identifying the pallet and the second digit identifying individual colony, label each point on the graph and illustrate changes in the pathogen abundance individual colonies throughout the sampling period.
(EPS)

S4 Fig Changes in KBV abundance in honey bee colonies throughout the sampling period. KBV abundance varied across the monitoring period in beekeeping operation 2, but was never detected in colonies from beekeeping operation 1. The natural log transformed values of the relative KBV abundance as determined by qPCR (y-axis) in honey bee samples (y-axis) were plotted by the date of sample collection (i.e. day of year, x-axis). Live honey bee samples were collected from colonies with varying colony health ratings (i.e., dead (black diamonds), weak (blue triangles), average (green circle), and strong (yellow square)). KBV abundance in colonies were plotted separately by beekeeping operation to indicate the effect of beekeeper on KBV abundance. Unique colony identifier numbers, with the first digit identifying the pallet and the second digit identifying individual colony, label each point on the graph and illustrate changes in the pathogen abundance individual colonies throughout the sampling period.
(EPS)

S5 Fig Changes in LSV2 abundance in honey bee colonies throughout the sampling period. LSV2 abundance was weakly correlated with date of sampling, but was greater in dead colonies. The natural log transformed values of the relative LSV2 RNA abundance as determined by qPCR (y-axis in honey bee samples were plotted by the date of sample collection (i.e. day of year, x-axis). Live honey bee samples were collected from colonies with varying colony health ratings (i.e., dead (black diamonds), weak (blue triangles), average (green circle), and strong (yellow square)). Unique colony identifier numbers, with the first digit identifying the pallet and the second digit identifying individual colony, label each point on the graph and illustrate changes in the pathogen abundance individual colonies throughout the sampling period.
(EPS)

S6 Fig Changes in LSV1 abundance in honey bee colonies throughout the sampling period. LSV1 abundance changed throughout the monitoring period, and was different between beekeeping operations. The natural log transformed values of the relative LSV1 RNA abundance as determined by qPCR (y-axis in honey bee samples (y-axis) were plotted by the date of sample collection (i.e. day of year, x-axis). Live honey bee samples were collected from colonies with varying colony health ratings (i.e., dead (black diamonds), weak (blue triangles), average (green circle), and strong (yellow square)). Unique colony identifier numbers, with the first digit identifying the pallet and the second digit identifying individual colony, label each point on the graph and illustrate changes in the pathogen abundance individual colonies throughout the sampling period.
(EPS)

S7 Fig Changes in *N*. *ceranae* abundance in honey bee colonies throughout the sampling period. *N*. *ceranae* abundance changed throughout the monitoring period and was predicted to be greater in strong rated colonies, compared to weak rated colonies. The natural log transformed values of the relative *N*. *ceranae* abundance as determined by qPCR in honey bee samples (y-axis) were plotted by the date of sample collection (i.e. day of year, x-axis). Live honey bee samples were collected from colonies with varying colony health ratings (i.e., dead (black diamonds), weak (blue triangles), average (green circle), and strong (yellow square)). Unique colony identifier numbers, with the first digit identifying the pallet and the second digit identifying individual colony, label each point on the graph and illustrate changes in the pathogen abundance individual colonies throughout the sampling period.
(EPS)

S8 Fig Changes in *L*. *passim* abundance in honey bee colonies throughout the sampling period. *L*. *passim* abundance was predicted to change throughout the monitoring period, but the abundance was greater in beekeeping Operation 1, than beekeeping Operation 2. The natural log transformed values of the relative *L*. *passim* abundance as determined by qPCR in honey bee samples (y-axis) were plotted by the date of sample collection (i.e. day of year, x-axis). Live honey bee samples were collected from colonies with varying colony health ratings (i.e., dead (black diamonds), weak (blue triangles), average (green circle), and strong (yellow square)). *L*. *passim* abundance in colonies were plotted separately by beekeeping operation to indicate the effect of beekeeper on *L*. *passim* abundance. Unique colony identifier numbers, with the first digit identifying the pallet and the second digit identifying individual colony, label each point on the graph and illustrate changes in the pathogen abundance individual colonies throughout the sampling period.
(EPS)

S9 Fig Changes in BQCV abundance in honey bee colonies throughout the sampling period. BQCV abundance changed throughout the monitoring period, and levels of abundance were higher in beekeeping Operation 2, than in beekeeping Operation 1. The natural log transformed values of the relative BQCV abundance as determined by qPCR in honey bee samples (y-axis) were plotted by the date of sample collection (i.e. day of year, x-axis). Live honey bee samples were collected from colonies with varying colony health ratings (i.e., dead (black diamonds), weak (blue triangles), average (green circle), and strong (yellow square)). BQCV abundances in colonies were plotted separately by beekeeping operation to indicate the effect of beekeeper on BQCV abundance. Unique colony identifier numbers, with the first digit identifying the pallet and the second digit identifying individual colony, label each point on the graph and illustrate changes in the pathogen abundance individual colonies throughout the sampling period.
(EPS)

S10 Fig Correlations between changes in frame count in response to individual pathogen abundances after accounting for sampling date. The residuals of the change in frame count (used as a proxy for changes in colony health rating), after accounting for the sampling date (i.e., “day of year”) (y-axis) were plotted relative to the natural log transformed values of the relative pathogen RNA abundance (x-axis) for (A.) DWV (B.) LSV1, (C.) LSV2, (D.) BQCV, (E.) KBV, (F.) *N*. *ceranae*, and (G.) *L*. *passim*. Live honey bee samples were collected from colonies with varying colony health ratings (i.e., dead (black diamonds), weak (blue triangles), average (green circle), and strong (yellow square)). The best-fit line (blue) surrounded by upper and lower standard error estimates (gray) indicates trends in the changes in frame count relative to pathogen abundance. After accounting for changes in frame counts over time, the only significant result was a negative relationship between KBV abundance and changes in frame count. Unique colony identifier numbers, with the first digit identifying the pallet and the second digit identifying individual colony, label each point on the graph.
(EPS)
