# Supplementary material for: Honey bee (Apis mellifera) colony health and pathogen composition in migratory beekeeping operations involved in California almond pollination
Source: PLoS One. 2017 Aug 17;12(8):e0182814. doi: 10.1371/journal.pone.0182814 (PMC5560708; doi:10.1371/journal.pone.0182814)
Supplement: S1 Table — (PDF) [file pone.0182814.s001.pdf]

Supplemental Table S1. Primers used in this study.

Genome / Gene Name	NCBI # GI #	Primer Name	Sequence (5'-3')	Product Size (bp)	Reference
**Ribosomal protein L8 (*Apis m.*)**	XM_393671.5<br>GI:571556074	Rpl8Fw<br>Rpl8Rev	TGGATGTTCAACAGGGTTCATA<br>CTGGTGGTGGACGTATTGATAA	121	Evans et al. (2006) Insect Mol Bio
**Lake Sinai virus 1 (LSV1)**	HQ871931<br>GI:335057596	qLSV1-F-2569<br>qLSV1-R-2743**	AGAGGTTGCACGGCAGCATG<br>GGGACGCAGCACGATGCTCA	174	Runckel, Flenniken (2011) PLoS One
**Lake Sinai virus 2 (LSV2)**	HQ888865<br>GI:335057589	qLSV2-F-1722<br>qLSV2-R-1947**	CGTGCTGAGGCCACGGTTGT<br>GCGGTGTCGATCTCGCGGAC	225	Runckel, Flenniken (2011) PLoS One
**Black queen cell virus (BQCV)**	AF183905<br>GI:8100530	qBQCVorf2F_6664<br>qBQCVorf2R_6805	TCCTCAAATCTGGAGCGAAC<br>GTATTCGCTGGCCGTAAAAC	141	Runckel, Flenniken (2011) PLoS One
**Deformed wing virus (DWV)**	AY292384.1<br>GI:31540603	DWVfw1165<br>DWVrev1338	CTTACTCTGCCGTCGCCCA<br>CCGTTAGGAACTCATTATCGCG	173	Chen et a. (2005) J Invert Path
**Kashmir bee virus (KBV)**	NC_004807.1<br>GI:30793779	KBV_F_4470<br>KBV_R_4581	TCGACAAGGACATGATCGAG<br>GAGCCACAAATGGCTTCTTC	111	Stoltz et al. (1995) J Apicult Res
Lotmaria passim / (Crithidia mellificae)	PRJNA78249<br>AHIJ01002555<br>Contig 777	qCrFw1<br>qCrRev1	TCCACTCTGCAAACGATGAC<br>GGGCCGAATGGAAAAGATAC	153	Runckel, Flenniken (2011) PLoS One
P. larvae	PRJNA30269	PL2-Fw<br>PL2-Rev	CGGGAGACGCCAGGTTAG<br>TTCTTCCTTGGCAACAGAGC	380	Marinez et al 2010;<br>Marinez et al 2011
M. plutonius	PRJDA73165	MelissoF<br>MelissoR	CAGCTAGTCGGTTTGGTTCC<br>TTGGCTGTAGATAGAATTGACAAT	796	Roetschi et al 2007;<br>Roetschi et al 2008
N. ceranae	DQ673615.1<br>GI:110293152	N ceranae F-4186<br>N ceranae R-4435	CGGATAAAAGAGTCCGTTACC<br>TGAGCAGGGTTCTAGGGAT	249	Chen et al. (2008) J Inv Path
