# Supplementary material for: Honey bee (Apis mellifera) colony health and pathogen composition in migratory beekeeping operations involved in California almond pollination
Source: PLoS One. 2017 Aug 17;12(8):e0182814. doi: 10.1371/journal.pone.0182814 (PMC5560708; doi:10.1371/journal.pone.0182814)
Supplement: S2 Table — Pathogen prevalence varied significantly between strong and average colony health ratings. Honey bee samples obtained from dead, weak, average, and strong colonies were tested for the presence of seven pathogens, including five viruses (i.e., DWV, BQCV, LSV1, LSV2, KBV), L. passim, and N. ceranae using PCR, and for Varroa destructor mites, which were counted as a pathogen when infestation levels were above the recommended treatment threshold of 3%. Total pathogen prevalence was determined by summing the number of pathogens detected in each sample. Honey bee colony population size was used as a proxy for colony health by counting the number of frames more than 2/3 covered with bees (i.e. weak < 7 frames, average = 7–12 frames, strong >12 frames covered with bees). Included in the table are the mean number of pathogens per colony strength rating, the standard error estimate of the mean, and the number of colonies per colony health rating within this cohort. This sample cohort was comprised of 52.2% strong, 37% average, 8.6% weak, and 2.2% dead colonies. (PDF) [file pone.0182814.s002.pdf]

S2 Table. Pathogen prevalence by colony health rating.

Pathogen prevalence varied significantly between strong and average colony health ratings. Honey bee samples obtained from dead, weak, average, and strong colonies were tested for the presence of seven pathogens, including five viruses (i.e., DWV, BQCV, LSV1, LSV2, KBV), *L. passim*, and *N. ceranae* using PCR, and for *Varroa destructor* mites, which were counted as a pathogen when infestation levels were above the recommended treatment threshold of 3%. Total pathogen prevalence was determined by summing the number of pathogens detected in each sample. Honey bee colony population size was used as a proxy for colony health by counting the number of frames more than 2/3 covered with bees (i.e. weak < 7 frames, average = 7-12 frames, strong >12 frames covered with bees). Included in the table are the mean number of pathogens per colony strength rating, the standard error estimate of the mean, and the number of colonies per colony health rating within this cohort. This sample cohort was comprised of 52.2% strong, 37% average, 8.6% weak, and 2.2% dead colonies.

colony health rating	mean pathogen prevalence	standard error (+/-)	sample size (n)
dead	6.50	1.50	2
weak	5.25	0.38	8
average	5.38	0.18	34
strong	5.95	0.13	48
