# Supplementary material for: Honey bee (Apis mellifera) colony health and pathogen composition in migratory beekeeping operations involved in California almond pollination
Source: PLoS One. 2017 Aug 17;12(8):e0182814. doi: 10.1371/journal.pone.0182814 (PMC5560708; doi:10.1371/journal.pone.0182814)
Supplement: S4 Table — Pathogen prevalence varied significantly by sampling event. Honey bee samples were obtained from two Minnesota based, commercially managed honey bee colonies, twice when colonies were located in California before and immediately after almond pollination and thus reflect the pathogen prevalence during the almond bloom, and three times after the almond pollination event when colonies were located in Minnesota. Pathogen prevalence was measured by totaling the number of pathogens detected via PCR in each sample. Included are the average number of pathogens per sampling event, the standard error estimate of the mean, and the number of colonies per colony health rating within this cohort. (PDF) [file pone.0182814.s004.pdf]

S4 Table. Pathogen prevalence by sampling event.

Pathogen prevalence varied significantly by sampling event. Honey bee samples were obtained from two Minnesota based, commercially managed honey bee colonies, twice when colonies were located in California before and immediately after almond pollination and thus reflective of the pathogen prevalence during the almond bloom, and three times after the almond pollination event when colonies were located in Minnesota. Pathogen prevalence was measured by totaling the number of pathogens detected via PCR in each sample. Included are the average number of pathogens per sampling event, the standard error estimate of the mean, and the number of colonies per colony health rating within this cohort.

sampling event	location	average pathogen prevalence	standard error (+/-)	sample size (n)
before	California	5.00	0.17	28
during	California	5.39	0.09	28
after 1	Minnesota	7.09	0.28	11
after 2	Minnesota	6.43	0.13	14
after 3	Minnesota	6.00	0.23	11
